# Polarity protein Par3L deletion causes chromosomal segregation defects and tumorigenesis

**DOI:** 10.1016/j.jbc.2025.110966

**Published:** 2025-11-20

**Authors:** Weili Zhang, Fengyan Dai, Yindan Hong, Chenyue Gao, Jiangchao Li, Lijun Dai, Yuxiang Liang, Yi Zhong, Yongliang Huo

**Affiliations:** 1 Guangzhou Municipal and Guangdong Provincial Key Laboratory of Protein Modification and Degradation, School of Basic Medical Sciences, Guangzhou Medical University, Guangzhou, China; 4 Experimental Animal Center, Guangzhou Medical University, Guangzhou, China; 2 Beijing No. 80 High School, Beijing, China; 3 Laboratory of Oncology and Immunology, School of Basic Medical Sciences, Guangdong Pharmaceutical University, Guangzhou, China; 5 Department of Urology, Guangdong Key Laboratory of Clinical Molecular Medicine and Diagnostics, Guangzhou First People’s Hospital, School of Medicine, South China University of Technology, Guangzhou, China

**Keywords:** cell polarity, cancer, chromosomes, Par3L, gene knockout

## Abstract

Apical-basal polarity is an intrinsic property of epithelial cells, which is governed by a set of conserved polarity proteins. Par3-like (Par3L), an ortholog of the classic polarity protein Par3, has been implicated in multiple diseases through genetic studies, but its biological functions are understudied. Here we found that Par3L deletion in mice lead to tumorigenesis in gastrointestinal track, prostates, and lungs. Par3L is also expressed in small subsets of cells in ovaries, testis, pancreas, brains, and kidneys, which did not show abnormal phenotypes in the Par3L KO mice. Further analysis of the gastrointestinal track in the Par3L KO mice found increased mitotic cells, which have significantly higher ratios of aberrant spindles compared to that of the WT mice. To delineate the potential mechanism, we tagged the endogenous Par3L coding sequence with a 3 x flag tag and identified the Par3L interacting proteins in intestine, kidneys, and pancreas. We found that Par3L interacts with proteins involved in chromatin remodeling and spindle assemblies, cytoskeleton and extracellular matrix, trafficking, metabolism, and mRNA processing. These data provide valuable information understanding the non-canonical polarity protein Par3L.

Epithelial cells cover the surface of the body and form tubular structures in ductal organs like kidneys, lungs, and pancreas *etc.* These cells are characterized by apical-basal polarity. They bear three distinct domains, the apical side facing the environment or the lumen, the lateral side stitching with neighboring cells by junctional proteins, and the basal side anchoring to the stroma. Three protein complexes coordinate the formation of the apical-basal polarity, including Crumbs/Pals1/Patj, Par3/Par6/Atypical protein kinase C (aPKC), and Scrib/Lgl/Dlg (1). Besides the morphologically distinct cellular domains, they also direct mitotic spindle orientation (2), asymmetric distribution of cell fate determinants during cell division (3, 4), and transport of cellular contents (5). This polarity regulating system is conserved in metazoans. Depletion of the polarity proteins in animals often results in mortality.

Polarity proteins are important players in mitotic spindle orientation. Polarity proteins Par3, Par6, aPKC, and Dlg were all implicated in the spindle orientation determination (6, 7). Actin network sets the foundation for spindle assembly and separation (8). During development and tissue homeostasis spindle orientation is strictly controlled. Most epithelial tissue development involves two stages. The first stage is the formation of primary integrated epithelial sheet or tubule structures. The second stage is the stratification or elongation of the primary epithelial structures to form functional tissues. In reality the two stages are not clearly separated, they may happen at the same moment in the same tissue. In tubular epithelial systems like renal tubules, airway tubes, and mammary ducts, the spindle poles are parallel to the basement membrane. This will cause two effects, namely, the tubules are lengthened when the spindles are aligned along the longitudinal axis or the tubules increase in diameter when the spindles are aligned within the transverse plane. The question is how the cells decide which direction to divide. In renal tubules the default spindle orientation seems to be within the transverse plane. When planar cell polarity signals emerge the spindle orientation switch to the longitudinal mode to elongate the tubules (9). In the airway tubes of the lung sprouty family genes, *fgf10* suppressor, regulate ERK1/2 signaling to control the spindle orientation and tissue shape (10). Normally cells in the tubular tissues do not divide perpendicular to the basement membrane. The reason is that aPKC at the apical cell cortex phosphorylates LGN (named after ten Leucine-Glycine-Asparagine tripeptides in its N-terminal region), which will disrupt the interactions between LGN and Gai. Thus, the LGN-NuMA complex is excluded from the apical cell cortex and relocate to the lateral sides, which direct the spindle orientation parallel to the basement membrane (2). The other possible mechanism is through the LGN linker signal. It has been shown in *drosophila* that LGN linker region is phosphorylated by Aurora A kinase to recruit Dlg for correct spindle orientation (7). Aurora A kinase was also shown in mouse mammary glands to direct spindle orientation (11). In skin- and neuro-epithelial systems, cells divide in two ways--symmetric division or asymmetric division. In symmetric division the spindle poles align parallel to the epithelial sheet, so the daughter cells will inherit similar cellular contents and extracellular environment. In this way the progenitor cells are amplified. In contrast, in asymmetric division the spindle poles align perpendicular to the epithelial sheet. As a result, the daughter cells inherit both cellular and extracellular cues differentially, which causes cell differentiation (12, 13). Studies showed that Insecutable is responsible for vertical spindle orientation and it dominates horizontal spindle orientation signals. In neuro-progenitor and skin progenitor cells over-expressed Insecutable concentrates at the apical cell cortex and recruits LGN to form an apical crescent. This would drive the cells to divide perpendicularly to the basement membrane (13, 14). When Insecutable is depleted by conditional knock-out in the neuro-progenitor cells mitotic spindles align horizontally, which should have been vertically otherwise (14). This suggests that the horizontal orientation signal is always present, whereas Insecutable overcomes this signal and turns the spindle to the vertical position. It was shown that Insecutable was recruited to the apical cell cortex by polarity protein Par3 (13). Planar cell polarity pathway may function in this process since it regulates neuroblast asymmetric division in *drosophila* and directs the longitudinal lengthening of mammalian tubule structure (9, 15, 16, 17). When LGN is depleted in neuro-progenitor cells mitotic spindle orientation is randomized (18). This observation indicates that LGN does not confer spindle orientation preference. Rather, LGN is an important regulatory component in the spindle assembly, which is regulated by the spindle orientation signals. For example, aPKC phosphorylation of LGN excludes the assembly from the apical cell cortex and Aurora A kinase phosphorylation of LGN drives the assembly to the lateral cell cortex (2, 7). A second regulatory mechanism on the spindle assembly may exist besides LGN. In the skin basal progenitor cells, NuMA can dissociate from apical LGN and re-direct the spindle to the lateral cell cortex when excessive asymmetric cell division happens (19). One study showed that NuMA bound directly to the cell membrane component Bind 4.1 to direct the spindle orientation (20).

In vertebrate systems polarity regulation is complex to accommodate complicated physiological and pathological activities. Genes evolved to have homologs and paralogs, and multiple splicing isoforms. Par3L is closely related to the classic polarity protein Par3 in vertebrates (21). Par3L shares similar protein domains with Par3, including three PDZ domains, and the aPKC binding domain. However, they showed differential binding capacity, namely, Par3 binds to both Par6 and aPKC, while Par3L does not bind aPKC, and only some splicing variant binds to Par6. Nonetheless, both Par3 and Par3L are localized at the tight junction of epithelial cells when ectopically expressed. Although the exact biological functions of Par3L are understudied, it has been implicated in numerous diseases, for example, diabetes, HIV infection, and cancer (22, 23, 24). We have previously found that Par3L is expressed in the cap cells of the developing mammary glands, although at very low levels. It binds to another polarity protein LKB1 to maintain cap cell stemness (25). In this study, we deleted the Par3L coding gene *pard3b* in mice and found that the gastrointestinal (GI) track, prostates, and lungs developed tumors. Further analysis found that the GI track has more mitotic cells, and significantly higher ratios of abnormal spindles compared to that in the WT mice. To explore the potential molecular mechanism, we inserted a 3xFlag tag at the 5-prime end of the *pard3b* gene to identify the Par3L interacting proteins in the GI track. We found that Par3L interacts with proteins involved in chromatin remodeling and spindle assembly. These data unveiled an unexpected role of the polarity protein Par3L in tumorigenesis of multiple organs, possibly through genome stabilization perturbation.

## Results

### Construction of Par3L KO mice

The Par3L coding gene *pard3b* has 23 exons and occupies 1.1 million base-pairs in the genome. To make the Par3L deletion, we first took a similar strategy to the Par3 depletion to excise the third exon (26). We designed two single guide RNAs (sgRNA), one in the third exon and the other in the third intron (Fig. S1*A*). CRISPR-associated protein 9 (Cas9) protein and the sgRNAs were mixed and microinjected into the C57BL/6 fertilized eggs, which were further transplanted into the surrogate females. After birth, mice were genotyped using PCR and the exact deletion was confirmed by sequencing (Fig. S1*B*). The F0 offspring were backcrossed onto C57BL/6 mice for five generations before analysis. Par3L was previously found to be highly expressed in the kidneys. We isolated kidneys from both WT control mice and the Par3L KO mice and stained Par3L. Par3L localized in the proximal tubule cells and the podocyte in the kidneys, however, no difference was observed between the WT mice and the Par3L KO mice (Fig. S1*C*).

We then tried to deplete Par3L by deleting the important domains. In previous studies, we showed that Par3L interacts with LKB1 through the intergenic sequences between PDZ2 and PDZ3 domain. In this way Par3L suppresses LKB1 kinase activity and maintain stemness of cap cells (25). To delete this region, we designed two sgRNAs, one in the exon 10 and the other in the exon 11 (Fig. S1*D*). F0 mice were PCR genotyped, and the deletion was confirmed by sequencing. A 32-kilobase fragment, including part of the exon 10, intron 10, and part of the exon 11 were excised (Fig. S1*E*). After 5-generation of backcrossing onto C57BL/6 mice, kidneys were stained for Par3L. We found that Par3L was still localized in the proximal tubule cells and the podocytes, with equal intensity in both WT mice and Par3L KO mice.

Finally, we tried to deplete Par3L by excising the promoter region. Two sgRNAs were designed flanking a 2-kilobase fragment from the promoter region to the first intron (Fig. S1*G*). Genotyping and sequencing confirmed two F0 mice with desired deletion, although both contained random insertions between the theoretical cutting sites (Fig. S1*H*). Par3L was highly expressed in the proximal tubule cells and podocytes in kidneys and epithelial cells in the pancreas of WT mice but absent in those cells of the Par3L KO mice. However, the depletion in the second Par3L KO mouse line was not complete, with weak signals in the proximal tubule cells and pancreatic epithelium (Fig. S1*I*). Quantitative PCR assay aligned with the immunofluorescent staining data. The Par3L mRNA levels in both Par3L KO mouse lines were significantly lower than that in the WT mice (Fig. S1*J*). These data were further supported by western blotting results, with significant lower amount of protein levels in the Par3L KO mouse lines than that of the WT mice (Fig. S1, *K* and *L*). These data demonstrate that the *pard3b* gene promoter region deletion successfully knocked-out Par3L in mice. Since the Par3L remnant persists in the second Par3L KO mouse line, we solely used the first Par3L KO mouse line for the remaining experiments.

### Par3L KO mice developed gastrointestinal tumors

**Figure 1:**
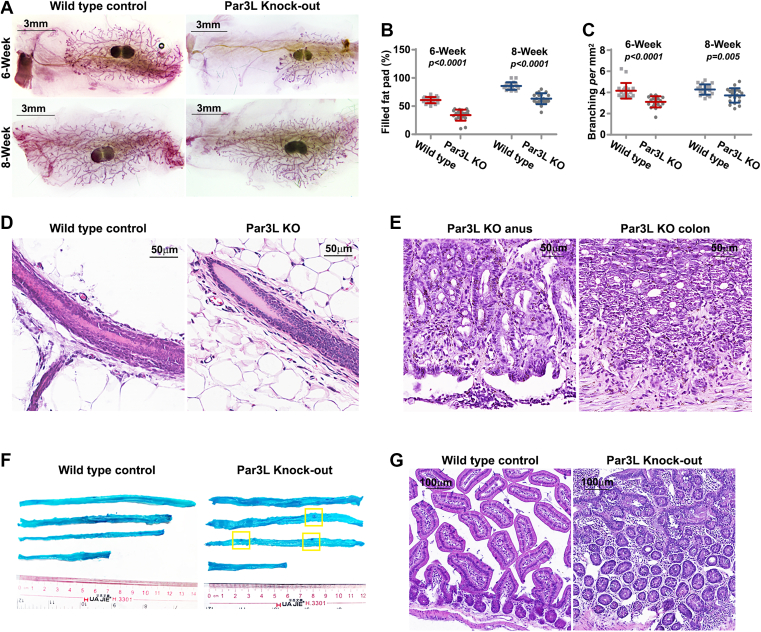
**phenotypes of Par3L KO mice**. *A*, mammary gland whole-mount images at pubertal and mature stages. *B*, mammary duct growth indicated by filled fat pad. *C*, mammary duct complexity indicated by branching points. *p* values were calculated by unpaired student *t* test compared to the WT controls. Data represent mean ± SD. n = 20. *D*, hematoxylin-eosin staining of mammary glands of 12-month-old mice. *E*, hematoxylin-eosin staining of gastrointestinal lesions of Par3L KO mice. *F*, methylene *blue* staining of the GI track of aged mice. G, microscopic analysis of hematoxylin-eosin-stained intestine of aged mice. GI, gastrointestinal.

We first analyzed the mammary glands for Par3L KO phenotypes. The mammary gland development is delayed in the Par3L KO mice compared to the WT mice (Fig. 1*A*). The ductal extension, evidenced by the fat pad filling ratio, is significantly suppressed by Par3L KO at both 6-weeks and 8-weeks after birth (Fig. 1*B*). Similarly, the complexity of the ducts, gauged by ductal branching, is significantly lower in the Par3L KO mice (Fig. 1*C*). However, the mammary glands eventually developed morphologically indistinguishable to those of the WT controls (Fig. 1*D*). Moreover, the female Par3L KO mice can conceive and lactate offspring comparable to the WT mice.

While we setup to analyze the aged females for mammary gland morphology, we found rare cases that the Par3L KO mice developed outgrowth at the anus and finally succumbed to death. We isolated the outgrowth and part of the colon for pathological analysis. Microscopic inspection found adenoma in both the anus and colon samples (Fig. 1*E*). To further confirm this finding, we isolated the entire GI track and subjected to methylene blue staining. Small outgrowths existed along the GI track of the Par3L KO mice, but not in the GI track of the WT controls (Fig. 1*F*). Microscopic analysis confirmed that these outgrowths are colorectal adenoma (Fig. 1*G*).

**Figure 2:**
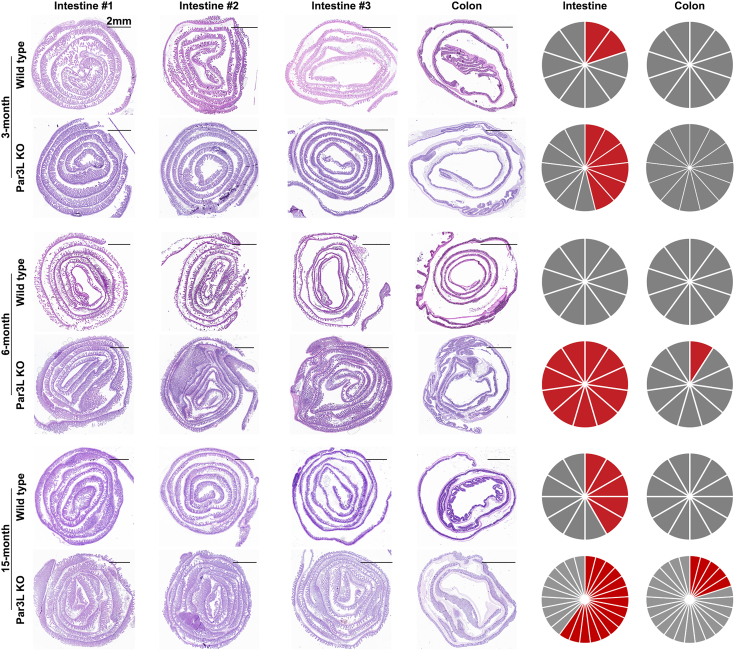
**GI track tumor incidence with time**. The slices of the pie chart represent mice analyzed and the *red slices* represent mice that developed tumors. The scale bar represents 2 mm.

We analyzed the penetration rate. Three groups of Par3L KO mice along with WT controls, including both males and females, were setup to analyze at different age. We collected the entire GI track at 3-, 6-, and 15-months’ time point for microscopic analysis. The Par3L KO mice developed adenoma in the GI track from 3 months. The penetration rate increased with age, while the WT mice remained normal at 6-months. At 15 months, both Par3L KO mice and the WT mice developed tumors in the intestine, while only Par3L KO mice had tumors in the colon (Fig. 2). Of note, all the adenoma we found was small, even though some showed clear signs of penetration into the stroma. These data clearly showed that Par3L depletion not only impaired the mammary gland development but also led to tumorigenesis in the GI track.

### Par3L knock-out results in tumorigenesis in lungs and prostates

**Figure 3:**
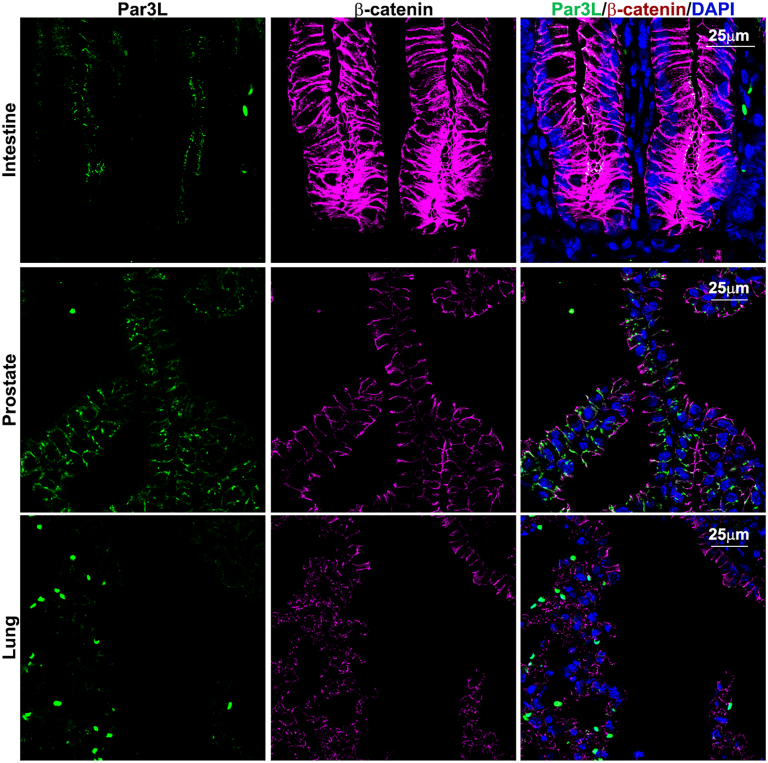
**Par3L expression patterns in different tissues determined by immunofluorescent staining**.

**Figure 4:**
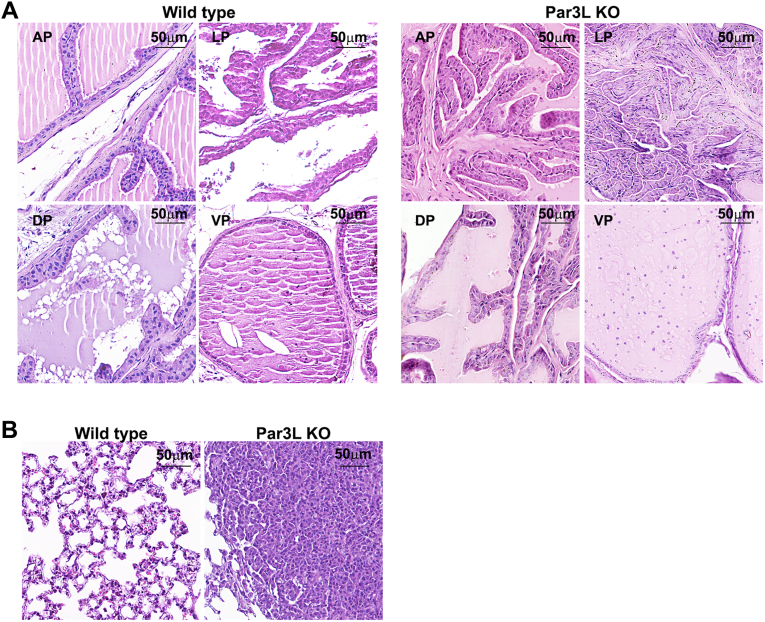
**microscopic analysis for tissues that express Par3L**. *A*, hyperplastic cells filled the prostate lumen in Par3L KO mice, but not in the WT control mice. *B*, tumors form in the lungs of Par3L KO mice, but not the WT control mice. Par3L, Par3-like.

The fact that Par3L depletion led to tumorigenesis in the GI track implies important biological functions of Par3L in tissues other than the mammary glands. With this in mind, we surveyed the expression pattern of Par3L in mice. Organs, including kidneys, pancreas, intestine, brain, prostates, lungs, testis, and ovaries were harvested from 12-week-old C57BL/6 mice for Par3L staining (Figs. 3 and S2). The expression levels in all tissues were low, consistent with the expression in cap cells of the mammary glands. The highest levels were seen in proximal tubule cells and podocytes of the kidneys and epithelial cells of the pancreas (Fig. 1*I*). In intestine, Par3L was localized at the junction of the cryptic cells, mutations of which may be responsible for the tumorigenesis. Par3L was also seen in the choroid plexus cells of the brain, follicular cells or corpus luteum cells of the ovary, and prostate epithelium. It was detectable in the lung epithelium, but at low levels (Figs. 3, and S2). We then collected organs from 12-month-old mice, including males and females, for microscopic analysis. No pathological changes in brain, ovaries, testis, kidneys, or pancreas were evident in the Par3L KO mice compared to the WT control mice (Fig. S3). However, the prostate showed hyperplasia with excessive epithelium filling up the lumen in half of the Par3L KO mice, while the prostate of the WT mice was clear in the lumen (Fig. 4*A*). Lung nodules are developed in 20% of the Par3L KO mice, but not the WT mice (Fig. 4*B*). These data indicate that Par3L is expressed in a small subset of cells in each organs examined and may have distinct biological functions, evidenced by the tumorigenesis in lungs, and prostates.

### Par3L knock-out mice have more abnormal mitotic cells in the GI track

**Figure 5:**
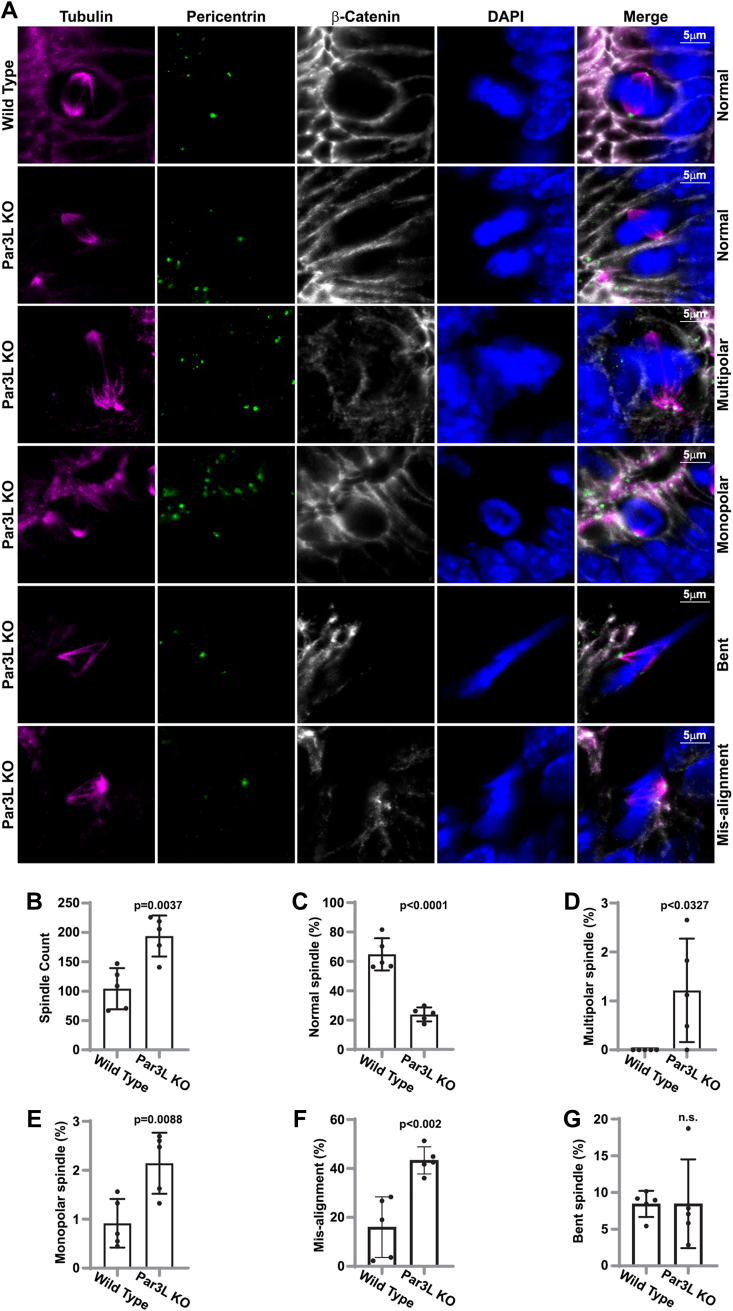
**mitotic cells in the intestine of Par3L KO and WT mice**. *A*, representative images of normal and defective mitotic cells. *B*, total numbers of mitotic cells in the duodenum of WT and Par3L KO mice. *C*, ratios of the mitotic cells bearing normal shaped spindle assembly. *D*, ratios of mitotic cells with multipolar spindles. *E*, ratios of mitotic cells with monopolar spindles. *F*, ratios of the mitotic cells with mis-aligned spindles. *G*, ratios of the mitotic cells with bent spindles. *p* values were calculated by unpaired student *t* test compared to the WT controls. Data represent mean ± SD. Data was acquired from five mice. Par3L, Par3-like.

When we analyzed the pathological slides of Par3L KO mice, we found high frequency of mitotic cells in the GI track. To verify this phenomenon quantitatively, we stained the duodenum of the intestine from both Par3L KO mice and the WT control mice and imaged all the mitotic cells (Fig. 5*A*). Indeed, the total number of mitotic cells in Par3L KO mice was significantly higher than that in the WT mice (Fig. 5*B*). Moreover, we found that over 60% of the mitotic cells in the WT mice had normal spindles, while about 80% mitotic cells in the Par3L KO mice contained defective spindles, including monopolar, multipolar, bent, unfocused, and mis-aligned spindles (Fig. 5*C*). Among them, the ratios of multipolar, monopolar, and mis-aligned spindles were significantly higher in the Par3L KO mice than those in the WT control mice (Fig. 5, *D*–*F*). The bent spindles were present in both the Par3L KO mice and the control mice, with similar ratios (Fig. 5*G*). Defective spindles often lead to genome instability that enables cells to acquire mutations during proliferation. These data suggest that Par3L KO causes GI track malignancy through genome instability.

### Par3L depletion promotes tumor phenotypes in adenocarcinoma cell CT26

**Figure 6:**
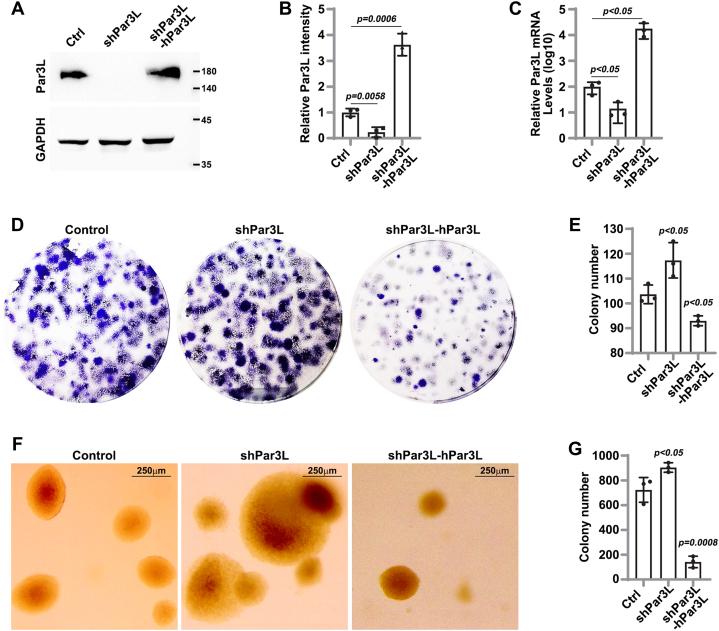
**Par3L deficiency enhances CT26 tumor phenotypes.***A,* representative images of western blotting assay. *B,* quantification and statistics for the western blotting assays. *C,* quantitative PCR assays to determine the mRNA levels of Par3L when Par3L shRNA or shPar3L-huPar3L is expressed in CT26 cells. *D,* colony forming assays for CT26 cells expressing the Par3L shRNA or shPar3L-huPar3L. Colony numbers were summarized in (*E*). *F,* softagar assay to test the anchorage-independent growth of CT26 cells expressing the Par3L shRNA or shPar3L-huPar3L. Tumorsphere numbers were summarized in (*G*). *p* values were calculated by unpaired student *t* test compared to the shRNA controls. Data represent mean ± SD. Data were acquired from three independent experiments. Par3L, Par3-like.

**Figure 7:**
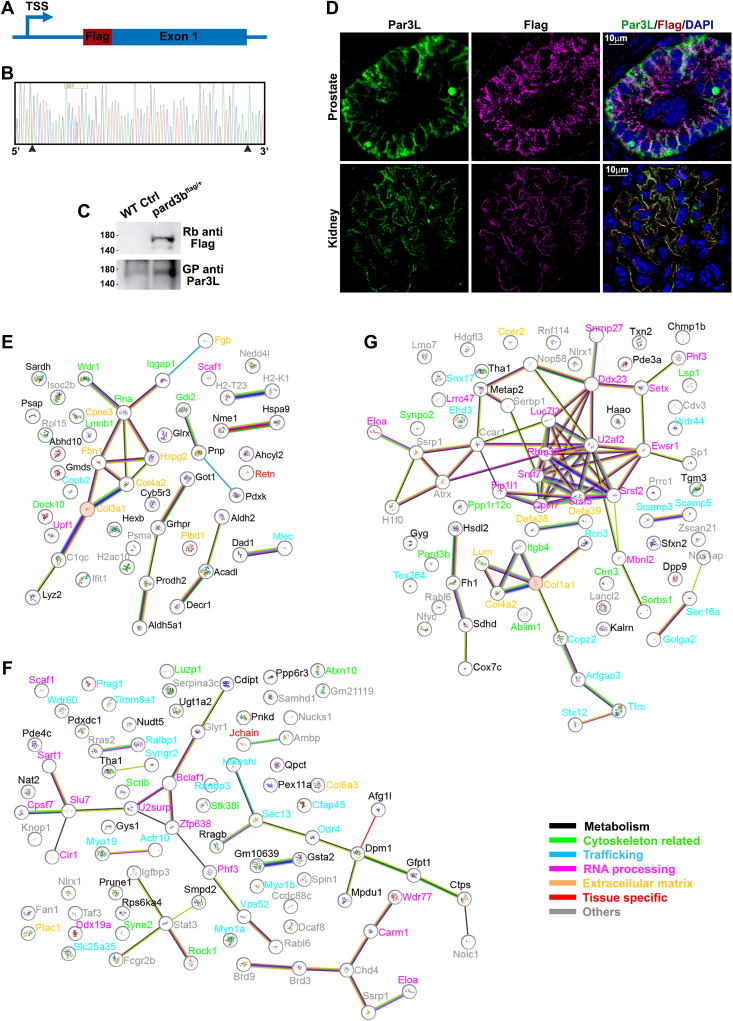
**Par3L interactors in mouse tissues.***A,* scheme to construct the transgenic mouse with flag tagged *pard3b* allele. *B,* sequencing data verified the flag tagged *pard3b* allele. *C,* western blotting assay showed that flag antibody and Par3L antibody cross reacted with a similar sized protein in the flag tagged Par3L transgenic mice. *D,* immunostaining demonstrated that Par3L antibody and flag antibody stained at the same localization in mouse prostates and kidneys. *E–G,* string analysis for Par3L interactors in the Pancreas (*E*), kidneys (*F*), and GI track (*G*). Proteins of similar functions were labeled in the same color. Par3L, Par3-like

To examine if Par3L deficiency results in tumor phenotypes, we knocked down Par3L in the mouse colon adenocarcinoma cell line CT26, which expressed low levels of Par3L (Fig. 7*A*). Stable expression of Par3L shRNA by lentivirus infection efficiently knocked-down Par3L in CT26 cells, evidenced by the decreased protein levels in western blotting assays (Fig. 6, *A* and *B*) and the decreased mRNA levels in quantitative PCR assays (Fig. 6*C*). The decrease in mRNA and proteins levels were rescued by overexpression of a non-targetable human Par3L (Fig. 6, *A*–*C*).

We assessed the tumor phenotypes of Par3L depleted cells. We found that Par3L knock-down slightly, but significantly, increased the colony-forming capability compared to the control shRNA. High levels of non-targetable Par3L expression significantly suppressed the colony forming efficiency (Fig. 6, *D* and *E*). In addition, Par3L knock-down improved the anchorage-independent growth of CT26 cells in soft agar assays. Similar to the colony forming assay, high levels of Par3L suppressed the tumorsphere formation (Fig. 6, *F* and *G*). Par3L expression in CT26 cells is extremely low, which may account for the modest increase in the colony formation and tumorsphere formation of Par3L knock-down cells. However, Par3L overexpression dramatically suppressed colony and tumorsphere formation, which strongly support Par3L as a tumor suppressor. Overall, these data demonstrate that Par3L depletion in CT26 cells enhanced tumor phenotypes.

### Par3L forms protein complexes with conserved functions in mouse tissues

Par3L KO resulted in tumorigenesis in intestine, prostates, and lungs, but no phenotypes in other organs like testis, ovaries, pancreas, or kidneys, which suggested that Par3L might function differentially across mouse tissues. To explore the possible signaling pathways that Par3L is involved, we tagged the endogenous *pard3b* gene with a 3xFlag at the 5′-end to uncover the Par3L protein complex in different tissues (Fig. 7*A*). The Flag insertion in the endogenous locus was verified by DNA sequencing (Fig. 7*B*). After backcrossing onto C57BL/6 mice, we examined the kidney protein lysate for Par3L and Flag using western blotting. Both Par3L and Flag antibodies cross-reacted with a protein of similar size in the Par3L^Flag/+^ mice, while the Par3L antibody, but not the flag antibody, resulted in a band in the WT mice (Fig. 7*C*). Consistent to these results, Par3L and Flag were co-localized on the membrane in kidneys and prostates of the Par3L^Flag/+^ mice (Fig. 7*D*). These results demonstrate that the *pard3b* locus was successfully tagged by the 3xFlag.

To dissect the composition of the Par3L protein complex we purified the Par3L protein complexes for mass spectrometry analysis. Three Par3L^Flag/+^ mice and three WT control mice were used to dissect kidneys, pancreas, and intestine. Protein lysates of these tissues were subject to the M2 flag antibody immunoprecipitation. Par3L interactors were identified by mass spectrometry (Table S1). For each tissue, we screened the proteins that are present in at least two of the three Par3L^Flag/+^ mice, and absent in all three WT control mice. We found 49 specific interacting proteins in the pancreas, 85 in the kidneys, and 76 proteins in the GI track (Fig. 7, *E*–*G*, and Table S2). In all three tissues, the Par3L associating proteins can be categorized into five groups based on their biological functions, namely, extracellular matrix, cytoskeleton, molecular trafficking and protein localization, metabolism, and mRNA processing. In each tissue, significant number of proteins are involved in various biological functions, for example, transcription regulation, signal transduction, *etc.*, which are grouped into others. In the pancreas, Par3L associated with Retn, a hormone that may suppress glucose uptake stimulated by insulin (27); and in the kidneys Par3L bound with Igj that is involved in the glomerular filtration (28). No tissue function specific proteins were observed in the GI track to interact with Par3L. In both GI track and kidneys Par3L interacted with proteins that are involved in chromatin remodeling and genome stabilization, for example, Brd3, Brd9, Chd4, Ssrp1, and Atrx (29, 30, 31, 32, 33). Among them, Luzp1, Spin1, Setx, and Atrx have been shown to regulate chromosome stability during mitosis (34, 35, 36, 37). This result supports the findings that Par3L KO increased abnormal mitotic spindles in the intestine. However, the fact that Par3L KO did not incur tumors in the kidneys implicates a second factor for tumorigenesis in the intestine. Tumorigenesis is a result of imbalance between cell proliferation and apoptosis, which favors proliferation over apoptosis. Epithelial cells in the GI track turnover relatively fast, usually within a week, to compensate for the sloughed cells in the villi. In contrast, renal epithelium, especially proximal tubule cells in which Par3L is highly expressed, does not proliferate under normal physiological conditions. As a result, the net accumulation of abnormal cells to noticeable lesions within the period of observation would be dramatically different.

**Figure 8:**
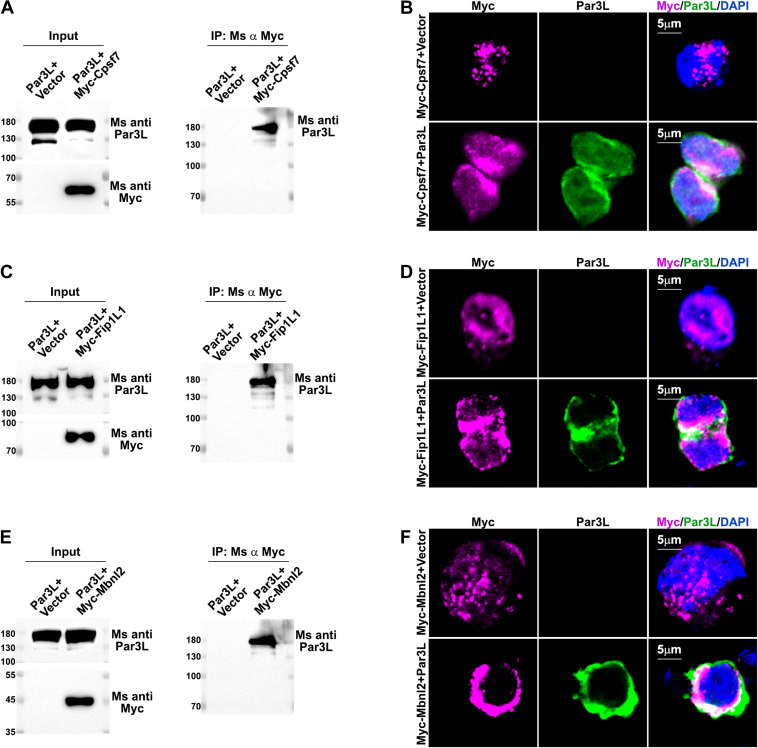
**Validation of Par3L binding proteins**. *A, C*, and *E,* Co-IP analysis of Par3L showed interactions between Par3L and Cpsf7, Fip1L1, and Mbnl2. *B, D*, and *F,* IF staining demonstrated that Cpsf7, Fip1L1, and Mbnl2 colocalized with Par3L. Par3L, Par3-like.

To assess the quality of the interactome, we employed protein docking to calculate the interaction area and free energy. The predicted protein structures of Par3L and its interactors in the GI track were extracted from the Alphafold database (38). Par3L and the interactors were docked using the fast Fourier transform algorithm by the GRAMM web-based docking tool (39). The interaction surface area and free energy of the yielded interaction models were calculated based on chemical thermodynamics, as a measure of the quality of the protein-protein assembly (40). A known Par3L interacting protein, LKB1, was used as a positive control. The positive control, LKB1-Par3L complex, has a free energy of −17.5 kcal/mol. Forty-six of the eight-one tested proteins have energy levels lower than the LKB1-Par3L complex, indicating a highly favorable interaction (Fig. S4 and Table S3). We further took three proteins that are not involved in known cell polarity functions, including Fip1L1, Mbnl2, and Cpsf7 for experimental validation using Co-IP analysis in HEK293T cells. All three proteins that we tested using Co-IP assays bind to Par3L (Fig. 8, *A*, *C*, and *E*). Moreover, we transformed Par3L or a vector control with Fip1L1, Mbnl2, or Cpsf7 into HEK293T cells for IF staining to test if Fip1L1, Mbnl2, and Cpsf7 colocalize with Par3L. Overexpression of Fip1L1, Mbnl2, or Cpsf7 causes excessive cell death and Par3L ameliorates this phenotype. Overexpressed Fip1L1, Mbnl2, and Cpsf7 distribute in the cytoplasm and nuclei. They partially colocalized with Par3L (Fig. 8, *B*, *D*, and *F*). These data demonstrate that the Par3L interactome is of high quality. In summary, Par3L interactomes vary greatly in different tissues, whereas they execute some conserved functions in each tissue. Among them, Par3L may be involved in chromatin remodeling and genome stability maintenance, which account for the tumorigenesis in the Par3L KO mice.

## Discussion

Par3L is a vertebrate homolog of the classic polarity protein Par3. It has been implicated in multiple diseases, yet the *in vivo* functions are unexplored. In this study, we constructed mouse models of Par3L KO using the Cas9 system. We find that Par3L KO significantly impairs mammary gland development in young mice, which reach the levels of the WT control mice with age. Surprisingly, Par3L KO leads to tumorigenesis in the GI track, prostates, and lungs. In the intestine of Par3L KO mice, more cells are in the mitotic stage than in the WT intestine. More importantly, Absence of Par3L results in significantly increased abnormal spindle assembly compared to the WT controls. The tumor suppression effect of Par3L is further confirmed using human colon cancer cell line CT26. Par3L depletion by shRNA enhanced the capabilities of colony-formation and anchorage-independent growth of colon cancer cells. To delineate the protein complex *in vivo*, we tagged the endogenous Par3L protein and performed immunoprecipitation followed by mass spectrometry. Par3L protein complexes in tissues are dramatically different, with similar functions. Altogether, these data depict a systematic map to understand the functions of Par3L *in vivo*.

The construction of Par3L KO mice was complicated by the gene structure. The CRISPR-Cas9 nuclease creates double strand breaks at the region of interest, which are repaired by non-homologous end joining mechanisms. Insertions or deletions generated in the repair process introduce frameshift and result in premature terminating codons in the protein coding sequences. These mutated transcripts are usually recognized and degraded by the nonsense-mediated mRNA decay machinery, leading to a dramatic decrease in the mRNA levels (41). However, Par3L has 23 exons and covers over one million base-pairs in the genome. Numerous splicing isoforms exist under physiological conditions, indicating the sequence complexity (21). When we targeted the third exon or the 10th and eleventh exons to knock-out Par3L, the protein persisted, evidenced by the immunofluorescent staining data using an antibody recognizing the last 14 amino acids of Par3L protein. Although genome editing can be readily verified by DNA sequencing, the protein levels are difficult to determine. The edited allele can encode various levels of proteins through cryptic start codons in the genome, or alternative splicing that skips the affected exons (42, 43, 44). In these circumstances, quantitative PCR assays may not be suitable to determine KO efficiency because primers at different regions may yield variable results. Western blot assays could be confusing as well since the splicing variants may result in multiple bands, especially for complex genes like Par3L. In our study, we used staining data as readout for gene deletion, which was further confirmed by quantitative PCR and Western blot assays. One concern is that Par3L deletion may perturb the expression of non-coding RNAs that are embedded in the intronic regions, since Par3L occupies over one million base-pairs in the genome. It will be great to overexpress the Par3L coding sequences for a rescue of the phenotypes attributed to Par3L deletion. Similar to the cancer phenotypes in mice, depletion of Par3L using an shRNA to interfere the Par3L mRNA in colorectal cancer cells enhanced the tumorigenicity. This highlights that the Par3L deletion is the dominant factor for the exhibited phenotypes.

Par3L interactomes differ dramatically in the three tissues tested, whereas they process similar biological functions. Par3L interacts with filamentous actin (F-actin) binding proteins and cytoskeleton remodeling proteins in kidneys, pancreas, and intestine. F-actin is organized to form a thin and flexible network or actin bundles beneath the plasma membrane to provide mechanical force for cell morphology maintenance and movement. Besides, the ordered actin structure provides a track for particle transportation powered by myosin proteins (45, 46). In this study, we found that Par3L interacts with multiple coatomer proteins, for example Copb2 in the pancreas, Copz2 in the intestine, and the COPII vehicle outer coat Sec13 protein. Moreover, myosin proteins were identified in the kidney. These results suggest that Par3L is involved in the F-actin mediated vehicle transportation. Recently, the actin structures were also found in the nucleus for nuclear expansion, chromatin organization, DNA replication and double strand break repair, etc (47, 48, 49, 50, 51, 52). In line with these functions, we identified histone and chromatin remodeling proteins, transcription factors and transcription co-factors, mRNA maturation proteins. Among them, Luzp1, Spin1, AtrX, and SetX have been shown to regulate chromosome stability, which align with the abnormal spindle assembly in Par3L KO mouse intestine (34, 35, 36, 37). One surprise is that Par3L protein complexes contain large amount of proteins involved in metabolism in all three tissues examined. Previously we showed that Par3L suppresses the activity of liver kinase B1 (LKB1), which phosphorylates the ATP sensor AMPK. The Par3L interactome in this study indicates that Par3L regulates vehicle transport for protein subcellular localization, which may also be responsible for the genomic encoded mitochondrial protein localization. Of note, we also identified tissue specific proteins in the Par3L protein complexes—Retn in the pancreas that inhibits glucose absorption, and Igj in the kidney that regulates Immunoglobulin (Ig)A nephropathy (27, 28).

One surprise was that Par3L KO results in tumorigenesis in multiple organs. Previously we found that Par3L interacts with LKB1 and suppresses LKB1 kinase activity (25). LKB1 heterozygous deficiency is a major cause for Peutz–Jeghers syndrome that features hamartomatous polyp formation in the gastrointestinal tract (53). LKB1 mutations were also found in lung cancer, breast cancer, and colon cancer (54, 55, 56, 57). Since Par3L suppresses LKB1 activity, one would expect that Par3L functions as a tumor gene, rather than a tumor suppressor gene. However, we found that Par3L deletion leads to tumorigenesis in lungs, prostates, and GI track. Specifically, Par3L KO causes mitotic spindle defects, which is a novel function for polarity proteins. Previously, polarity proteins were found to direct spindle orientation during development and tissue homeostasis. Epithelial cells divide in certain directions, either along the basement stroma or along the apical-basal axis. This is coupled with the polarity cues provided by polarity proteins such as Par3 and aPKC *etc.* Normally, the Leucine-Glycine-Asparagine repeat protein (LGN) binds to the GDP-bound Gai that is anchored on the cell cortex through myristoylation (58, 59). LGN recruits the nuclear mitotic protein apparatus (NuMA) protein to the plasma membrane that is released to the cytoplasm when the nuclear envelope breaks down (60). This Gai-LGN-NuMA protein complex is crucial in spindle positioning, which dictates the division direction and daughter cell position. However, Gai is evenly distributed on the plasma membrane, which does not provide polarity cues. In contrast, the LGN protein is polarized through multiple mechanisms. Par3 restricts aPKC to the apical membrane of epithelial cells to phosphorylate the LGN, which dissociate LGN from apical Gai and exclude it from the apical domain. Cells with Par3 depletion preferentially divide along the apical-basal axis (2). On the lateral side, LGN can bind to E-cadherin, Afadin, Dlg, and Annexin A1 to position the spindles parallel to the basement stroma (61, 62, 63, 64, 65). This precise control of spindle orientation is critical for development, cell fate determination, and tissue homeostasis (3, 13, 66, 67, 68). Disruption of polarity has also been attributed to cancer progression, but mainly due to their functions in various signal transduction processes. Par3 loss dramatically promotes cancer cell spread to form massive lung metastasis. Par3 depletion activates JAK/Stat pathway in an aPKC dependent manner, which in turn induces MMP9 to degrade the extracellular matrix (69). Par3 depletion also aberrantly activates Rac1 through the release of its guanine exchange factor Tiam1. This leads to reduced cortical actin at the zonula junctions and modification of cadherin dynamics (70). Similarly, the lateral polarity complex disruption also causes cancers in multiple tissues. Scribble protein can promote tumorigenesis through a variety of pathways. Displacement of scribble activates mTORC1 through PTEN inactivation in basal-like breast cancer, and depletion of scribble in prostate tissues activates MAPK pathway to initiate prostate cancer (71, 72). In another study, Zhan *et al.* have shown that Scribble loss cooperates with Myc to inhibit activation of apoptosis and induce mammary tumorigenesis (73). The LLGL2 protein in the scribble complex was demonstrated to interact with the leucine transporter Slc7a5 to enhance the leucine uptake by ER + breast cancer cells, which promote cell proliferation and confer cancer cell drug resistance (74). Par3L deletion, however, disturbed the spindle assembly and affected the chromosome segregation into daughter cells during mitosis, which lead to tumorigenesis in the GI track. An intriguing observation is that Annexin A1 and LGN not only function as spindle orientation regulators, their deregulation in cells also results in multiple spindle-poles and disturbs chromosome segregation. It would be interesting if Par3L also regulates spindle orientation. However, Par3L is distributed evenly on the cell cortex, which would disrupt the oriented spindles dictated by polarity cues, contrary to the Par3 function.

Altogether, we generated Par3L KO mice using CRIPSR/Cas9, which resulted in tumorigenesis in intestine, lungs, and prostates. The Par3L KO intestine features high levels of mitotic cells, especially those with abnormal spindle assembly. The interactome analysis reveals that Par3L possesses conserved functions across different tissues. The deletion of Par3L possibly led to intestinal tumors due to defects in genome stability maintenance during cytokinesis. These results illustrate important roles of polarity protein Par3L in conserved cellular functions.

## Experimental procedures

Mice were generated using CRISPR/Cas9 system. For global KO of the Par3L coding gene, *pard3b*, guide RNAs were mixed with Cas9 protein and injected into fertilized eggs of C57BL/6 mice (Table 1). A 10 kb DNA fragment flanking the start codon was used to generate the Flag tagged Par3L allele. The Flag tag was built in-frame after the start codon, and two LoxP sites in the promoter region and the first intron, respectively. Transgenic mice were screened using PRC combined with sequencing. Positive mice were backcrossed to C57BL/6 for five generations before analysis. All mice were hosted in a specific antigen free facility with a 12-h light and 12-h dark cycle. C57BL/6 mice were purchased from Charles River through their Chinese distributor. All animal experiments were reviewed and approved by the Institutional Animal Care and Use Committee of Guangzhou Medical University.

**Table 1:** Oligo sequences

Name	Sequences	Comment
SgPromoter	tgttagatttgcaacccaact	Guide RNA for Exon 1 KO
SgExon1	ggtccttacaactccgccatc	Guide RNA for Exon 1 KO
Sg Exon10	ggtcattgcccgccaggaa	Guide RNA for Exon 10–11 KO
Sg Exon11	gattgttatgctctctccc	Guide RNA for Exon 10–11 KO
SgExon3	tggctgcatttaaacctgt	Guide RNA for Exon 3 KO
SgIntron3	tgctgctggagctcaggta	Guide RNA for Exon 3 KO
P3L Pro For	agagaaacgcctgtgagcccact	Genotyping primers
P3L I1 Rev	gccaatcaatgctcaaactccaag	Genotyping primers
Par3L For	ttcgagctggaccacttgctta	qPCR primers
Par3L Rev	tccgagcctggtaatactcctt	qPCR primers
GAPDH For	ggtgctgagtatgtcgtggagt	qPCR primers
GAPDH Rev	tgctgacaatcttgagtgagttg	qPCR primers

Par3L, Par3-like.

For mammary gland wholemount imaging, the tissue was spread onto glass slides and fixed in Carnoy’s fixative (60% ethanol, 30% chloroform, and 10% glacial acetic acid) for 2 hours before rehydration in ethanol gradually. Tissues were stained in Carmine-Alum (0.2% carmine and 0.5% aluminum potassium sulfate) for 1 h at room temperature and washed in water. Tissues were dehydrated in ethanol gradually and clarified in TO reagent before sealing in resin for imaging. For methylene blue staining, the gestor-intestinal track was washed in PBS and fixed in 4% paraformaldehyde (PFA) for 1 hour on filter paper. Tissues were stained with 0.2% methylene blue and 4% PFA for 10 seconds and washed in 4% PFA before imaging. For immunofluorescent staining, 5micron paraffin sections were de-waxed in TO reagent and rehydrated in ethanol before antigen retrieval using a pressure cooker. Tissues were blocked (10% western blocking reagent, 5% goat serum, and 0.3% Triton X-100) for 1 hour and incubated in primary antibodies overnight at 4 °C. Tissues were washed extensively in 0.3% Triton X-100/PBS and incubated in secondary antibodies for 1 hour at room temperature. Slides were finally sealed in anti-fade mounting medium for imaging. Detailed information of antibodies was listed in Table 2.

**Table 2:** Reagent information

Reagent	Manufacture	Cat. #	[Conc.]
GP anti Par3L	Homemade		1:100
Ms anti Par3L	Santa Cruz	Sc-398761	1:600
Ms anti Tubulin	Proteintech	66031-1-Ig	1:500
Ms anti β-Catenin	BD	610,154	1:500
Rb anti Myc	Proteintech	16286-1-AP	1:1000
Ms anti c-Myc	Santa Cruz	sc-40	1:1000
Rb anti DYKDDDDK	Thermo	PA1-984B	1:1000 for WB 1 μg/ml for IF
Gt anti Ms AF Plus 488	Invitrogen	A32723	1:1000
Gt anti Ms AF Plus 555	Invitrogen	A32727	1:1000
Gt anti Ms AF Plus 647	Invitrogen	A32728	1:1000
Gt anti Rb AF Plus 488	Invitrogen	A32731	1:1000
Gt anti Rb AF Plus 555	Invitrogen	A32732	1:1000
Gt anti Rb AF Plus 647	Invitrogen	A32733	1:1000
Gt anti GP AF 488	Invitrogen	A-11073	1:1000
Gt anti GP AF 555	Invitrogen	A-21435	1:1000
Gt anti Ms IgG, HRP	Invitrogen	A16072	1:5000 for WB
Gt anti Rb IgG, HRP	Invitrogen	A16104	1:5000 for WB
Gt anti GP, HRP	Invitrogen	A18769	1:5000 for WB 1:500 for IHC
DAPI (Hoechst 33,342)	Sigma	B2261–25 MG	10 μg/ml
Protein A/G magnetic beads	MCE	HY-K0202	
DAB	Genetech	GK347010	
Hematoxylin Eosin	Leagene	DH0006	
Fluoromount-G	Southernbiotech	0100–01	

Par3L, Par3-like.

To quantify the mitotic cells in the intestine, duodenum of both Par3L KO mice and C57BL/6 mice were immuno-stained. Ten-micron paraffin sections were stained for tubulin, b-catenin, pericentrin, and DAPI and analyzed on a Leica SP8 confocal microscope. All mitotic cells were recorded for analysis. Pericentrin was used to determine the numbers of mitotic spindle poles, and tubulin was used to identify the shapes and distribution of spindles.

For Quantitative PCR analysis, kidney RNA was extracted by Tri reagent (Sigma) and reverse transcribed using the Takara PrimeScript RT Reagent Kit according to the manufacture’s instruction. Genes of interest were quantified using the ChamQ Sybr qPCR master mix (Vazyme). Primer sequences are listed in Table 1.

For mass spectrometry analysis, intestinal and kidney tissue samples, which had been frozen in liquid nitrogen after collection, were ground in liquid nitrogen into fine powder and placed in 15 ml tubes containing sufficient IP lysis buffer (25 mM Hepes pH 7.4, 150 mM NaCl, 0.5 mM EDTA, 5 mM MgCl_2_, 0.5% TritonX-100, 2 mM DTT) to facilitate tissue lysis. IP lysis buffer was supplemented with protease and phosphatase inhibitors. Fifty micro-liter protein extract was stored as the input control, and the rest (500 μl per sample) was rotated head-to-toe for 2 h at 4 °C with anti-Flag M2 magnetic beads that had been blocked with 0.5% BSA. The magnetic beads were washed three times with Co-IP High-Salt buffer (25 mM Hepes pH 7.4, 450 mM NaCl, 0.5 mM EDTA, 5 mM MgCl_2_, 0.5% TritonX-100, 2 mM DTT) and then three times with Co-IP Wash buffer (25 mM Hepes pH 7.4, 150 mM NaCl, 0.5 mM EDTA, 5 mM MgCl_2_, 2 mM DTT). Pancreatic tissue was minced and then digested with 5 ml digestion medium (2 mg/ml Collagenase A, 15 mg/ml Hyaluronidase, 20 μg/ml DNase I, in Dulbecco's Modified Eagle's Medium (DMEM/F12). Epithelial cells were collected by centrifugation and subjected to IP with M2 magnetic beads. Proteins were digested on beads in 100 μl trypsin and 2 μl 2-Hydroxypropyl-β-cyclodextrin (100 mM) for 17 h at 37 °C. Peptides were analyzed on Q Exactive HF-X and identified by MaxQuant (1.6.17.0) with a false positive rate less than 1%. Three WT control and three Par3L KO mice were analyzed. Peptides of a protein appeared in at least two of the Par3L KO mice, but not the control mice, were considered positive hits.

HEK293T and CT26 were originally obtained from ATCC. Cells were cultured in high-glucose DMEM supplemented with 10% fetal bovine serum, 1% glutamax, and 1% penicillin/streptomycin at 37 °C with 5% CO_2_ unless specified. For soft agar assays, 1.2% low-melting agarose was pressure sterilized and cooled down to 42 °C before mixed with equal volume of 2 × cell culture medium. The diluted agarose was dispensed in tissue culture plate and solidified. Cells were mixed with 0.7% low-melting agarose at 42 °C and plated onto the agarose base for solidification. Cells were fed every 3 days and cultured for 2 to 4 weeks at 37 °C with 5% CO_2_. At the end of culture, colonies were imaged and those with a diameter greater than 50micron were counted. For colony formation assays, cells were well trypsinized, seeded in tissue culture plate, and cultured for 2 weeks at 37 °C with 5% CO_2_. Cells were fixed in 4% PFA and stained with 0.005% crystal violet. After imaging, colonies greater than 50 cells were counted.

For lentivirus production, 15 million HEK293T cells were seeded in 15 cm dishes 24 h before transfection. Lentiviral plasmid, packaging plasmid (psPAX2), and coat protein plasmid (pMD2.G) were mixed at a molar ratio of 3:2:1 and transfected into cells using EZ Trans Reagent (Li Lab). Medium was replaced with DMEM/2.5% FBS 12 h after transfection. Forty-eight hours after transfection, supernatant was collected and filtered through a 0.45 μm filter after brief centrifugation. The virus supernatant was mixed with equal volume of PEG8000 and precipitate overnight at 4 °C. Virus was precipitated by centrifugation at 4000 rpm, dissolved in 200 μl DMEM, aliquoted and stored at −80 °C. To establish stable cell lines, five thousand cells were infected in 20 μl media at an MOI of 20. Cells were cultured for 2 weeks and sorted for GFP- or RFP-positive cells using fluorescence-activated cell sorting on a BD LSR-II Flow Cytometer.

For Western blot assay, cells were lysed using RIPA lysis buffer (150 mM NaCl, 50 mM Tris Base, 1% Sodium deoxycholate, 0.1% SDS, 1% TritonX-100, pH 7.2) supplemented with protease inhibitors and phosphatase inhibitors for 10 min on ice. The extracted protein was quantified using a BCA Protein Assay Kit and de-natured at 98 °C in loading buffer. Protein samples were resolved in 8 to 12% gels and then transferred onto a nitrocellulose membrane. The membrane was incubated in a blocking solution of 5% milk in Tris-buffered saline with 0.05% Tween-20 (TBS-T) at room temperature for 1 h. Afterward, the membrane was incubated overnight at 4 °C with primary antibodies. The next day, the membrane was washed and incubated with HRP-conjugated secondary antibodies, at a dilution of 1:5000 in 5% milk in TBS-T. Bound antibodies were visualized using SuperSignal West Femto Maximum Sensitivity Substrate or SuperSignal West Pico PLUS Chemiluminescent Substrate. Images were captured using the ChemiDocMP Imaging System. Quantification of the images was performed using ImageJ (https://imagej.net/software/fiji/). Signals were normalized to the loading controls. All experiments were performed at least three times independently.

For protein docking, protein structures were downloaded from alphafold database at https://alphafold.ebi.ac.uk/. Par3L and its interactor were docked using a web-based tool at https://gramm.compbio.ku.edu/request based on the fast Fourier transform algorithm. We used a free docking method and choose the best docking model for presentation using PyMOL (ver. 3.0.3, https://www.pymol.org/). The docking model was further calculated for free energy and interaction area at https://www.ebi.ac.uk/pdbe/pisa/using auto processing mode.

## Data availability

All data are available upon request.

## Supporting information

This article contains supporting information.

## Conflict of interest

The authors declare that they have no conflicts of interest with the contents of this article.
