# Supplementary material for: Polarity protein Par3L deletion causes chromosomal segregation defects and tumorigenesis
Source: J Biol Chem. 2025 Nov 20;302(1):110966. doi: 10.1016/j.jbc.2025.110966 (PMC12767855; doi:10.1016/j.jbc.2025.110966)
Supplement: Supporting information [file mmc1.docx]

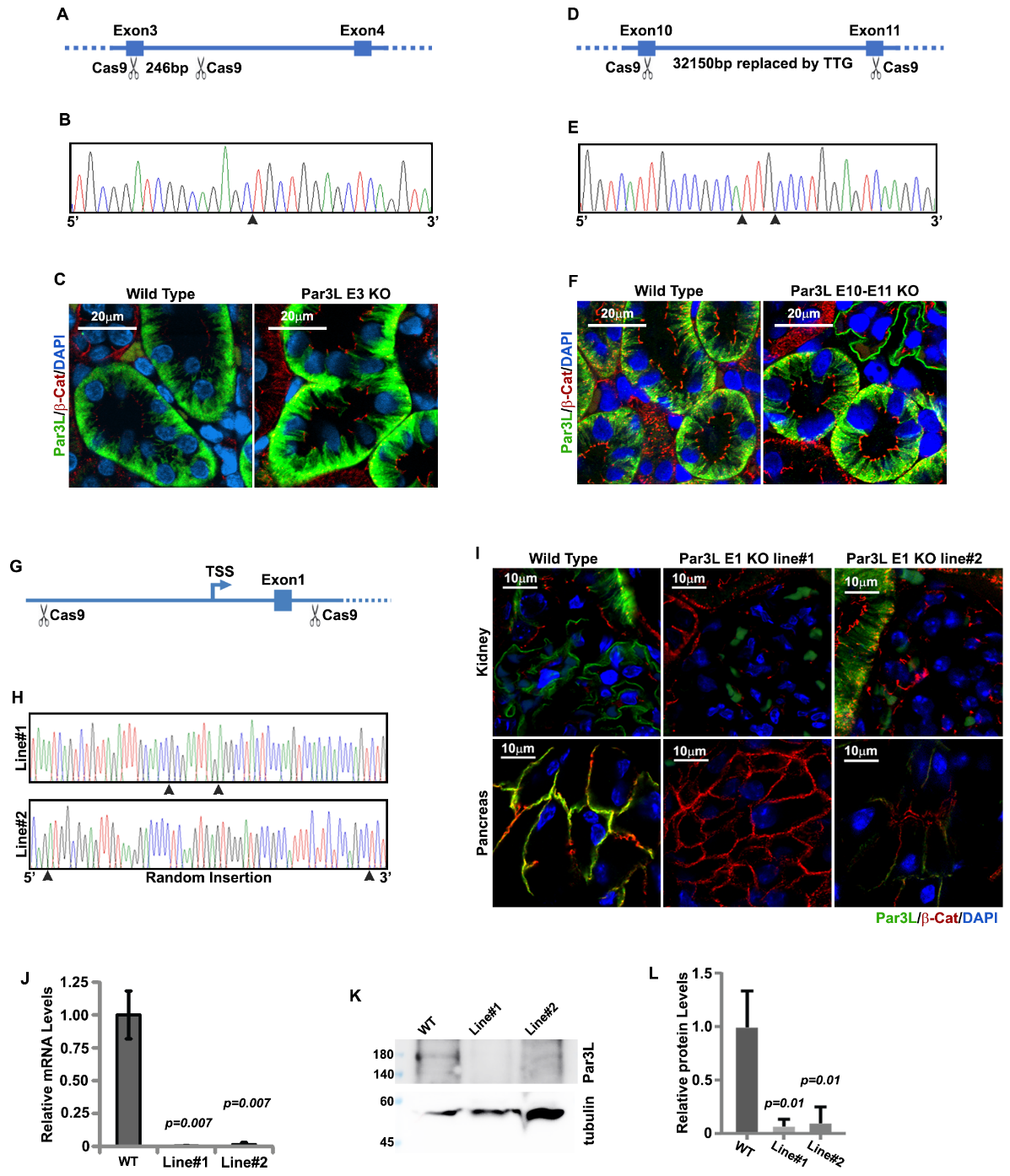


**Figure S1. generation of Par3L KO transgenic mice.** *A*, Scheme of Par3L exon3 deletion. *B*, sequencing results of the F0 Par3L KO mice. *C*, Par3L staining in kidneys of the Par3L exon3 KO mice. *D*, Scheme of Par3L exon10-11 deletion. *E*, sequencing results of the F0 Par3L KO mice. *F*, Par3L staining in kidneys of the Par3L exon 10-11 KO mice. *G*, Scheme of Par3L exon1 deletion. *H*, sequencing results of the F0 Par3L KO mice. *I*, Par3L staining in kidneys and pancreas of the Par3L exon10-11 KO mice. *J*, mRNA levels of Par3L determined by quantitative PCR. *K-L*, protein levels of Par3L determined by western blot assays. The intensity of protein bands in (*K*) were quantified using ImageJ and summarized in (*L*). P values were calculated by unpaired student *t* test compared to the wild-type controls. Data represent mean ± SD. n=3.


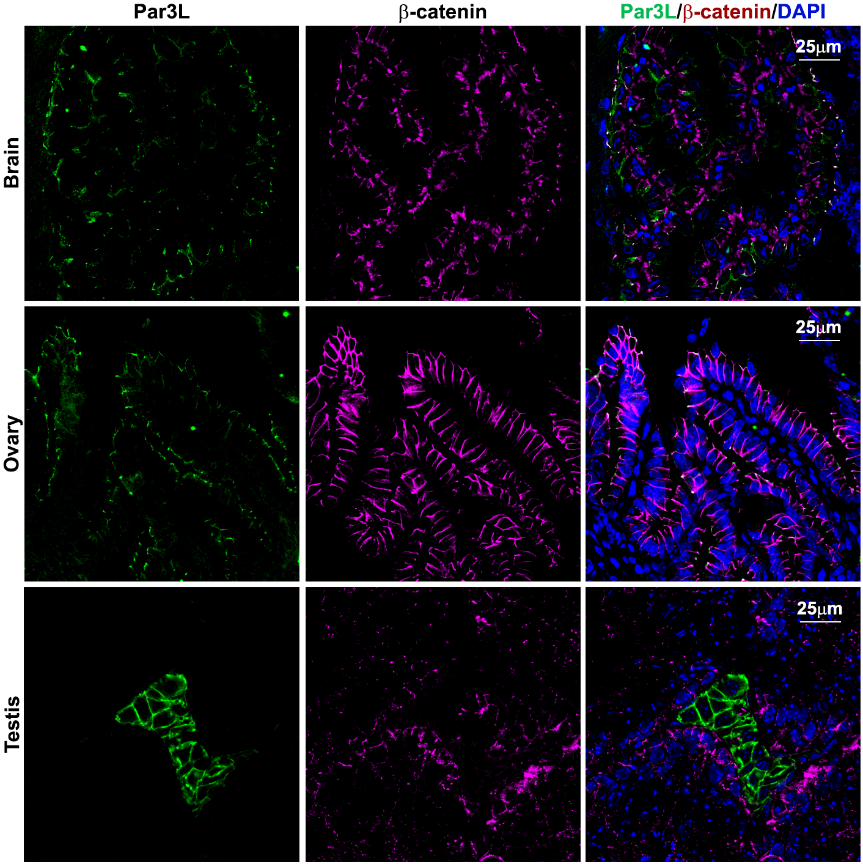


**Figure S2. Par3L expression in brain, ovary, and testis determined by immunofluorescent staining.**


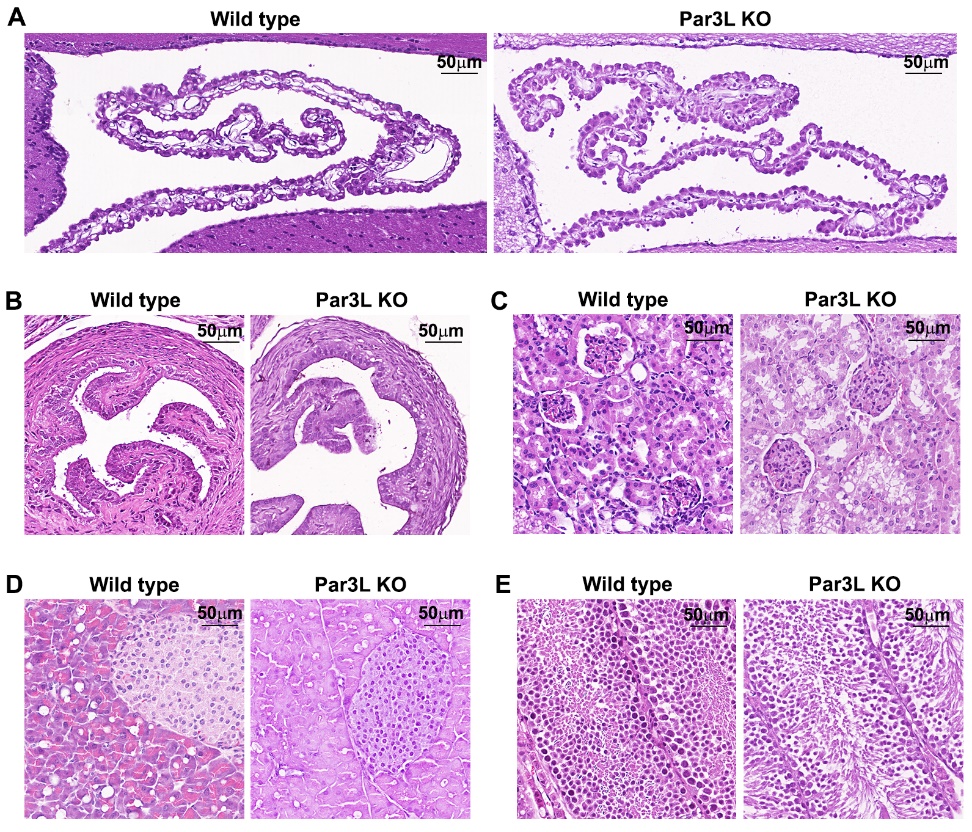


**Figure S3. microscopic analysis for tissues that express Par3L.** Images are choroid plexus (*A*), Ovaries (*B*), kidneys (*C*), pancreas (*D*), and testis (*E*). No specific phenotypes were observed in the Par3L KO mice compared to the wild-type control mice.


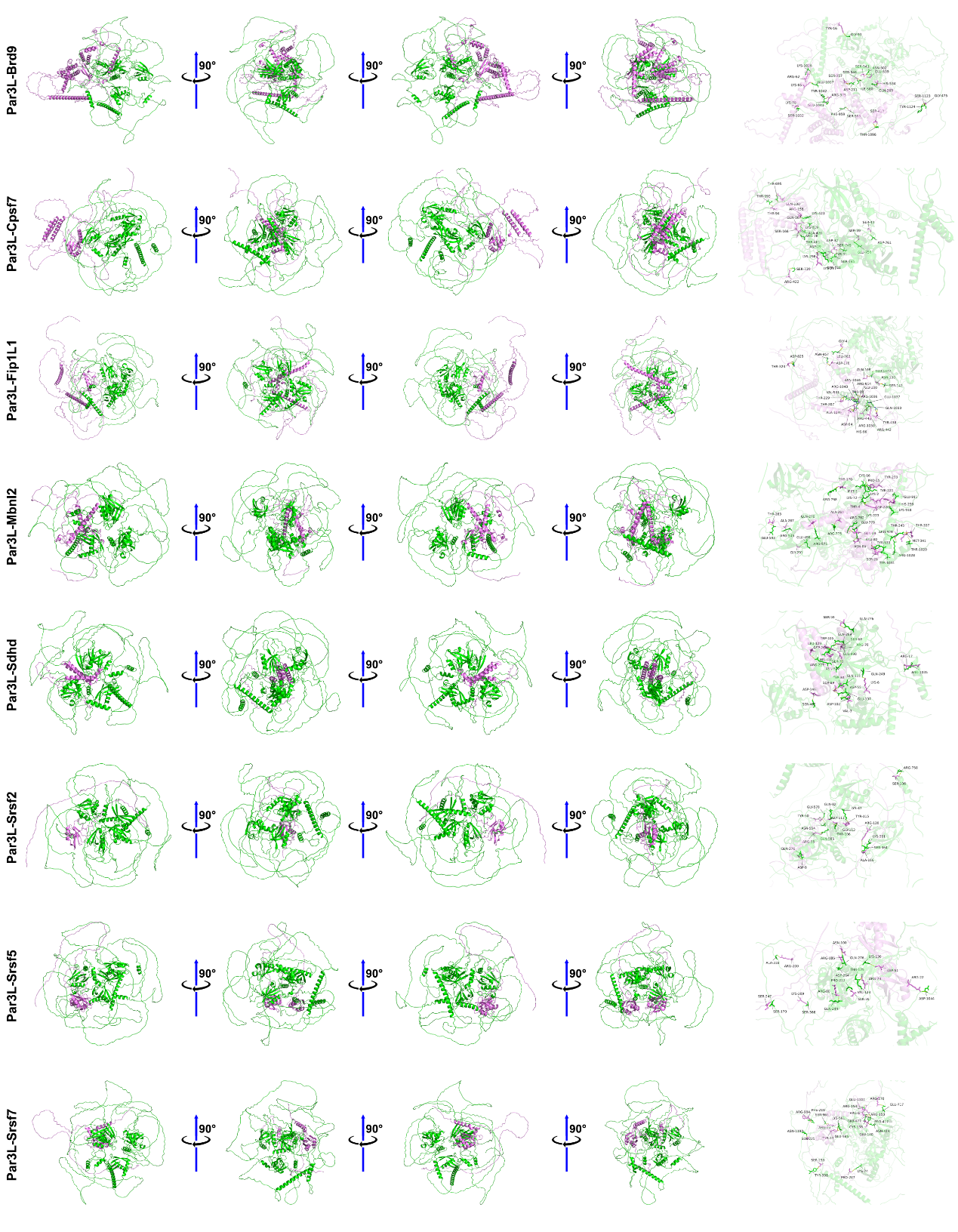


**Figure S4. Molecular docking models of Par3L and its interactors.**
